# Diabetes-Related Services and Programs in Small Local Public Health Departments, 2009-2010

**Published:** 2011-12-15

**Authors:** Akiko S. Hosler, Nur Zeinomar, Kofi Asare

**Affiliations:** Department of Epidemiology and Biostatistics, University at Albany (SUNY) School of Public Health; University at Albany (SUNY), Rensselaer, New York; University at Albany (SUNY), Rensselaer, New York

## Abstract

**Introduction:**

Local health departments (LHDs) vary in their capacity to perform public health services by the size of population they serve. Little is known about the extent of emerging primary prevention activities at small LHDs. The objectives of this study were to describe various diabetes-related patient care and primary prevention services offered by small LHDs (those serving a population of less than 150,000) and explore factors associated with the diversity of these services.

**Methods:**

During 2009 through 2010, we interviewed directors of a nationally representative sample of small LHDs by telephone to obtain information about staff structure, diabetes services, and partnerships. We obtained data for demographic characteristics and health status of the population from secondary sources. We analyzed the number of patient care services and primary prevention programs through multivariate regression analyses.

**Results:**

Fifty-eight small LHDs completed the survey, a response rate of 81%. Most (n = 47) had at least 1 diabetes-related patient care service; referral to diabetes specialists was the most frequently identified service (n = 44). Nearly half of small LHDs also engaged in obesity prevention for adults (n = 26) or children (n = 26), but only 7 had a diabetes prevention program. Diversity of patient care services was positively associated with the proportion of the population that was rural, time commitment of a certified diabetes educator, and total staff size. Diversity of primary prevention programs was positively associated with intensity of collaboration with the state diabetes program and total staff size and inversely associated with the proportion of racial/ethnic minorities in the jurisdiction.

**Conclusion:**

Most small LHDs function as a link to local diabetes care services. Staff capacity, collaboration with the state health department, and local population factors appear to influence the diversity of diabetes-related services at small LHDs.

## Introduction

Diabetes is a serious chronic disease affecting nearly 24 million Americans (1) and incurs an annual cost of $218 billion (2). Reducing the burden of diabetes is a major public health goal in the United States (3). At the state level, state health departments' diabetes prevention and control programs (DPCPs) collaborate with the Centers for Disease Control and Prevention (CDC) to implement comprehensive diabetes public health programs (3-5). At the local level, however, a coordinated effort to address the increasing burden of diabetes is lacking (6).

Local health departments (LHDs) vary widely in their structural capacity to perform public health services. Previous studies have found that a large population size is one of the most consistent predictors of public health performance (7-10). Some large metropolitan LHDs, such as those in New York City and Los Angeles County, have demonstrated their exceptional capacity to assess local diabetes burden and implement unique programs to address disparities in diabetes care (11-13). Furthermore, these large LHDs have adjusted to the epidemiological transition from communicable to chronic disease and developed a wide array of primary prevention programs for chronic diseases (6).

Little is known about the extent of diabetes-related programs at LHDs serving less populous jurisdictions (hereafter, "small LHDs"). Previous studies suggest that small LHDs still serve as traditional providers of patient care services to address secondary and tertiary prevention of diabetes, but they may not have resources to incorporate emerging primary prevention activities (14-16). A recent study investigated factors associated with diabetes-related programs at LHDs nationwide, but the scope of this study was limited only to diabetes screening and obesity prevention programs (17). We do not know what organizational and population factors are associated with being able to offer more diabetes-related services.

Most LHDs serve jurisdictions with a population of less than 150,000 (18). Additionally, rural residents, who are overrepresented in small LHD jurisdictions, are often at increased risk for diabetes and obesity. Excluding high-achieving large metropolitan LHDs from analysis would produce a more realistic picture of typical LHDs and portray their challenges. The objectives of this study were to describe diabetes-related patient care and primary prevention services offered by small LHDs and to explore factors associated with the diversity of these services.

## Methods

### Sampling

We obtained a database with information on every known LHD in the United States from the RAND Corporation (19). RAND constructed its database by identifying health departments in each state from a comprehensive list compiled by the National Association of County and City Health Officials (NACCHO) (20), through extensive Internet searches, and through direct contacts with local and state public health officials (19). A total of 2,459 LHDs that had working telephone systems were retained in the database (19).

Data on jurisdictions' total population were obtained from the 2000 US Census. We defined a small LHD as one serving a population of less than 150,000. This cutoff represented the first quartile of US population served by LHDs, systematically eliminating large metropolitan LHDs (19). We identified 2,071 small LHDs and stratified them into 8 strata, using the combination of 2 population sizes (<50,000 and 50,000-149,999) and 4 US regions (Northeast, South, Midwest, and West, as defined by the 2000 US Census). We conducted a sample-size estimation analysis to identify sample size that would allow us to compare 2 subgroups of small LHDs. Significance was set at *P* < .05, a power of 80%, and a prevalence ratio of 2.0. A total sample size of 62 was determined to be sufficient. Factoring in the expected nonresponse rate of approximately 10%, we randomly selected 72 small LHDs (9 LHDs per stratum).

### Telephone survey

We developed a 21-item structured telephone survey questionnaire for directors of the small LHDs. Presence or absence of specific diabetes-related services were determined with a series of dichotomous questions. From a review of literature (14,16,17,21) and informal interviews with a convenience sample of staff at small LHDs, we created a list of services that were most likely to be offered at small LHDs. Selected services included 5 patient care services addressing secondary and tertiary prevention of diabetes (diabetes screening, referral to local diabetes care specialists, diabetes self-management education including nutrition education, visiting nurse services for adults with diabetes, and school-based services for children with diabetes), and 3 programs related to primary prevention (obesity prevention for adults, obesity prevention for children and adolescents, and type 2 diabetes prevention). We also asked about capacity to conduct diabetes surveillance, using a specific example of estimating number of people with diabetes in the jurisdiction.

We assessed information about collaboration with the state health department's DPCP and other organizational partners by dichotomous questions. If collaboration was reported, probing questions were used to collect brief descriptions of the collaboration and information on the availability of funding. We assessed staff structure by self-reported total number of staff in full-time equivalent (FTE) positions and their academic credentials. If there was a certified diabetes educator (CDE), we asked whether his or her employment status was full-time or part-time.

In April 2009, we pilot-tested the questionnaire with 5 small LHDs not selected for the sample and confirmed that the questionnaire was clear and the protocol was appropriate. A graduate student assistant was trained as a telephone interviewer. Data collection was conducted from July 2009 through June 2010. Because of the H1N1 influenza outbreak, many directors of small LHDs were unavailable for interview for several months. We placed at least 5 calls on different days of the week and different hours of the day before we categorized them as nonrespondents. Three directors of small LHDs faxed their responses. The University at Albany institutional review board approved this study.

### Secondary data

For the small LHDs that completed the telephone survey, we collected data about population characteristics from the 2000 US Census SF-3 files. Information regarding types of jurisdiction (subcounty district, single county, or multiple counties), rural population (farm and nonfarm rural populations combined by the census designation), racial/ethnic minorities (nonwhite race or Hispanic ethnicity), and residents below the federal poverty level were obtained. The Census Bureau provided county-level prevalence estimates for residents younger than 65 years who had no health insurance coverage (22). CDC provided county-level prevalence estimates for diagnosed diabetes and obesity (defined as a body mass index of ≥30 kg/m^2^) among adults (23). For small LHDs representing subcounty jurisdictions, we used the prevalence estimate for the entire county as a surrogate.

### Data analysis

We calculated design weights to adjust for uneven sampling fractions and nonresponses across the 8 strata. The design weight equaled the ratio of the expected sample size based on the overall sampling fraction and the actual sample size in each stratum. The design weights enabled each small LHD to represent its proportion in the sampling frame. The weights ranged from 0.32 to 2.5, with a mean of 1.00. All values in this study were weighted.

We conducted descriptive analyses to depict organizational and population characteristics, availability of diabetes-related services, and current collaboration. We measured the percentage of public health departments in our sample that fell above or below the mean or median value for the United States for such characteristics as proportion of rural residents.

We defined diversity of diabetes-related services as a total number of individual services or programs offered. We coded the CDE variable as 0 for no CDE, 1 for a part-time CDE, and 2 for a full-time CDE. Similarly, collaboration with the state diabetes program and other organizations were coded as 0 for no collaboration, 1 for collaboration without funding, and 2 for collaboration with funding. To examine factors associated with diversities of patient care services and primary prevention programs, we conducted multivariate ordinary least-squares regression analyses. We used the forward stepwise deletion method to eliminate nonsignificant factors. We report the final models' standardized regression coefficients and their significance (*P* values). SPSS for Windows version 18.0 (SPSS, Inc, Chicago, Illinois) was used for statistical analysis.

## Results

Of the 72 small LHDs we sampled, 58 completed the survey, a response rate of 81%. Respondents had nearly identical distributions of population-size categories and regions, and a similar distribution of jurisdiction types compared to all small LHDs in the nation after weight adjustment (data not shown).

Forty-one of the sampled small LHDs were serving a single-county jurisdiction, and 44 were serving a jurisdiction with a population less than 50,000 (Table 1). Forty-four were serving a jurisdiction with a proportion of rural population higher than the national average. Most small LHDs were also serving a jurisdiction with a prevalence of diagnosed diabetes and obesity higher than the respective national median. In terms of departmental staff structure, 17 had fewer than 6 FTEs, and another 12 had 6 to 10 FTEs. Thirty-eight of the small LHDs had staff with a master's degree, but only 1 LHD had a full-time CDE, and remaining 3 LHDs were served by part-time CDEs.

Overall, 47 small LHDs had at least 1 diabetes-related patient care service. The most frequently offered diabetes-related patient care service was referral to local diabetes care specialists (n = 44) (Table 2). Visiting nurse services for adults with diabetes was the second, offered by 19 small LHDs. For primary prevention programs, obesity prevention programs for adults and for children/adolescents were offered at close to half of small LHDs. These programs included school-based physical activity and nutrition programs, after-school exercise programs for children, community-based walking/exercise programs, and worksite wellness programs for adults. Type 2 diabetes prevention was offered by 7 small LHDs. Programs for diabetes prevention primarily focused on increasing physical activity and promoting weight loss, combined with diabetes awareness activities. Overall, 35 small LHDs had at least 1 primary prevention program. Only 10 small LHDs reported having capacity to conduct diabetes surveillance.

Collaboration with the state health department's DPCP was reported by only 5 small LHDs, of which 4 had funding. More small LHDs (n = 22) reported collaboration with other organizations for diabetes-related projects, although only 6 had collaborations that came with funding. The most frequently reported partners were hospitals, followed by community-based organizations, local health coalitions, other small LHDs, and universities or medical schools (data not shown).

A greater number of patient care services was significantly associated with a larger proportion of rural population, greater time commitment of a CDE, and more FTEs (Table 3). Additionally, having more primary prevention programs was associated with greater collaboration with the state diabetes program and larger number of FTEs but a smaller proportion of racial/ethnic minorities in the jurisdiction.

## Discussion

We found that most small LHDs had at least 1 diabetes-related patient care service, but the proportion of those having any primary prevention program was lower. Patient referral was by far the most frequently mentioned diabetes service, illustrating that linking local residents to needed health care services is an important function of small LHDs. Diabetes care through visiting nurses and school health programs, diabetes screening, and diabetes education were also offered, but at lower frequencies.

Obesity prevention programs were offered by approximately half of small LHDs, compared with 56% of LHDs of all sizes that reported the presence of an obesity prevention program in the 2005 NACCHO national survey (17). Type 2 diabetes primary prevention programs were offered by only a small number of LHDs. The scarcity of diabetes primary prevention was not surprising, given the newness and difficulties of implementing diabetes primary prevention in public health (24). Some of these challenges include limited funding, a lack of coordination across disease-oriented programs, the large at-risk population, and scarcity of evidence-based practices (24). Also expected was the low percentage of small LHDs with diabetes surveillance capacity. Previous studies have found that smaller population size was associated with decreased capacity and performance of surveillance (9,10).

Our findings indicate that small LHDs mostly function as a link to local health care services and that they are transitioning to include obesity prevention in their inventory of services. They lag behind, however, in the more technically demanding area of surveillance and meeting the challenges of primary prevention of type 2 diabetes.

The number of patient care services was positively associated with the proportion of rural population, time-commitment of a CDE, and total FTEs. Rural residents face multiple barriers to access affordable health care; thus, small LHDs' role as providers of a range of diabetes patient care is likely greater with a higher proportion of rural residents in the jurisdiction (14). The importance of having a CDE for detection, care, and education was also reinforced by this finding, although hiring and retaining a CDE at a small rural LHD could be a major challenge (14). This finding was also congruent with that of a previous study that demonstrated that the presence of a health educator was associated with the availability of diabetes screening programs at LHDs nationwide (17).

The diversity of primary prevention programs was associated with increased collaboration with the state DPCP, total FTEs, and lower proportion of racial/ethnic minorities. The previous nationwide study also reported that the presence of an obesity prevention program was associated with more staff, external collaboration, and state funding (17). For primary prevention programs, technical and financial support from the state health department appeared to be particularly relevant. A case study of North Carolina LHDs demonstrated that successful implementation of primary prevention programs at LHDs was linked to the state health department's effort to provide technical assistance, training opportunities, and an evaluation framework to the LHDs (21). Funding for obesity prevention at the local level could come from various state health department programs, including those for obesity, cardiovascular disease, and the Special Supplemental Nutrition Program for Women, Infants and Children. Collaboration with the state DPCP therefore may imply the LHD's access to and involvement with other health department programs. The negative association with racial/ethnic minority proportion may be partly due to a lack of culturally and linguistically appropriate primary prevention models for minority populations. Further research is needed to explore this issue.

As discussed earlier, surveillance was one of the weakest performance areas of small LHDs. Even for the small LHDs that reported having diabetes surveillance capacity, their resources and technical levels were not sufficient to produce information that could influence program decisions. This does not seem to contradict our finding that having multiple diabetes-related services were not associated with the prevalence of diagnosed diabetes or obesity.

This study has limitations. We did not collect fiscal and budgetary information from the small LHDs because we determined that this type of information was too complex and sensitive to collect through a telephone survey. We also limited the length of the questionnaire to reduce respondent burden; therefore, we did not collect some other potentially important information, such as background of the agency executive and relationship with the local board of health. The diabetes-related services we assessed were those that were most likely to be offered at small LHDs, and they did not represent the universe of potential diabetes-related services. The estimates of diagnosed diabetes and obesity we used were derived from the extrapolation of state-level health telephone survey data; thus, they have their own intrinsic limitations (23). Finally, the cross-sectional design of this study did not allow us to examine the direction of associations.

Despite these limitations, by systematically excluding high-achieving large metropolitan LHDs, this study presents a realistic picture of typical LHDs in the United States. The use of a telephone survey allowed us to collect information directly from a sample of small LDH directors. Because the sample was selected and adjusted to be nationally representative, findings from this study are generalizable to small LHDs across the United States. The supplemental data from various sources enabled us to incorporate potentially important population information such as estimates for the prevalence of diabetes, obesity, and uninsured population, in the analyses.

In summary, we found that small LHDs primarily function as providers of diabetes patient care services rather than as providers of primary prevention services. We anticipate this situation will likely continue, given the persistent and increasing difficulties of accessing health care in many rural communities. All small LHDs should be prepared to make an additional effort to incorporate primary prevention programs because the burden of diabetes and obesity is projected to increase even more (25,26). Our study indicated that staff capacity, collaboration with state health departments, and certain local population factors are needed to diversify program activities. In addition, capacity for diabetes surveillance needs to improve so that program decisions can be guided by the need of the populations served. Currently, most chronic disease surveillance activities are conducted at the state level (27). Strengthening collaboration with state health departments is likely to improve small LHDs' technical capacity to conduct their own surveillance activities or access local diabetes surveillance information provided by the state DPCP.

## Figures and Tables

**Table 1:** Characteristics of Jurisdiction, Population Served, and Staff Structure of Small Local Health Departments^a^

**Characteristic**	No. of Local Health Departments (n = 58)^b^
**Jurisdiction characteristic**	
**Type of jurisdiction**	
Subcounty district	12
Single county	41
Multiple counties	5
**Population served**	
<50,000	44
50,000-149,999	14
**Racial/ethnic minorities, %^c^**	
≤30.9	48
>30.9	10
**Residents below poverty level, %^c^**	
≤12.4	43
>12.4	15
**Rural population, %^c^**	
≤20.5	14
>20.5	44
**Uninsured residents aged <65 y, %^c^**	
≤17.3	41
>17.3	17
**Prevalence of diagnosed diabetes among adults, %^d^**	
≤8.0	19
>8.0	39
**Prevalence of obesity (BMI ≥30 kg/m^2^) among adults, %^d^**	
≤26.3	23
>26.3	35
**Staff structure**	
**Total full-time equivalents**	
1-5	17
6-10	12
>10	29
**Staff with a master's degree**	
0	20
1-3	27
>3	11
**Staff with CDE qualification**	
0	54
Part-time	3
Full-time	1

Abbreviations: CDE, certified diabetes educator; BMI, body mass index.

a Health departments that serve <150,000 people.

b Percentages are weighted.

c The cutoff value represents the mean value for total US population.

d The cutoff value represents the median value for all US states and territories.

**Table 2:** Diabetes-Related Services, Programs, and Current Collaborative Partners at Small Local Health Departments^a^

**Description**	No. of Local Health Departments (n = 58)^b^
**Patient care service**	
Referral to local diabetes care specialists	44
Visiting nurse services for adults with diabetes	19
Diabetes screening	15
Diabetes self-management/nutrition education	13
School-based services for children with diabetes	6
Have at least 1 personal care service	47
**Primary prevention**	
Obesity prevention for adults	26
Obesity prevention for children and adolescents	26
Type 2 diabetes prevention	7
Have at least 1 primary prevention program	35
Have diabetes surveillance capacity	10
**Collaborative partner for diabetes-related services**	
Any state health department DPCP	5
DPCPs with funding	4
Any other organization	22
Other organizations with funding	6

Abbreviation: DPCP, diabetes prevention and control program.

a Health departments that serve <150,000 people.

b Percentages are weighted.

**Table 3:** Diversity of Patient Care Services and Primary Prevention Programs Among Small Local Health Departments^a^

Characteristic	Diversity of Patient Care Services (*R* ^2^ = .308)		Diversity of Primary Prevention Programs (*R* ^2^ = .531)	

	Multivariate Β^b^	*P* value	Multivariate Β^b^	*P* value
Percentage of racial/ethnic minorities	NA^c^	NA^c^	−.258	.01
Percentage of rural residents	.365	.002	NA^c^	NA^c^
No. of full-time equivalent staff	.275	.03	.249	.02
Time-commitment of staff CDE	.282	.02	NA^c^	NA^c^
Collaborates with state DPCP	NA^c^	NA^c^	.319	.009

Abbreviations: NA, not applicable; CDE, certified diabetes educator; DPCP, diabetes prevention and control program.

a Health departments that serve <150,000 people.

b Values are weighted.

c Not applicable because this variable is not included in the model.
